# Modeling Suicidality with Multimodal Impulsivity Characterization in Participants with Mental Health Disorder

**DOI:** 10.1155/2023/8552180

**Published:** 2023-08-05

**Authors:** Nidal Moukaddam, Bishal Lamichhane, Ramiro Salas, Wayne Goodman, Ashutosh Sabharwal

**Affiliations:** ^1^Menninger Department of Psychiatry, Baylor College of Medicine, Houston, TX, USA; ^2^Electrical and Computer Engineering, Rice University, Houston, TX, USA; ^3^The Menninger Clinic, Houston, TX, USA; ^4^Center for Translational Research on Inflammatory Diseases, Michael E. DeBakey VA Medical Center, Houston, TX, USA

## Abstract

**Introduction:**

Suicide is one of the leading causes of death across different age groups. The persistence of suicidal ideation and the progression of suicidal ideations to action could be related to impulsivity, the tendency to act on urges with low temporal latency, and little forethought. Quantifying impulsivity could thus help suicidality estimation and risk assessments in ideation-to-action suicidality frameworks.

**Methods:**

To model suicidality with impulsivity quantification, we obtained questionnaires, behavioral tests, heart rate variability (HRV), and resting state functional magnetic resonance imaging measurements from 34 participants with mood disorders. The participants were categorized into three suicidality groups based on their Mini-International Neuropsychiatric Interview: none, low, and moderate to severe.

**Results:**

Questionnaire and HRV-based impulsivity measures were significantly different between the suicidality groups with higher subscales of impulsivity associated with higher suicidality. A multimodal system to characterize impulsivity objectively resulted in a classification accuracy of 96.77% in the three-class suicidality group prediction task.

**Conclusions:**

This study elucidates the relative sensitivity of various impulsivity measures in differentiating participants with suicidality and demonstrates suicidality prediction with high accuracy using a multimodal objective impulsivity characterization in participants with mood disorders.

## 1. Introduction

Suicide is one of the leading causes of death across age groups [2]. Increasing evidence shows that suicide is not associated with any specific psychiatric diagnosis [3]. Many characteristics associated with lifetime suicide risk have been identified, but there are no systematic or reliable short-term predictors of suicidal behavior. Furthermore, while suicide attempts are currently the most useful predictor for suicide, over half of the suicides occur on the first attempt [4]. The long-term propensity to have bouts of suicidal behavior is identified as suicidal behavior disorder (SBD). It may be a latent trait that predicts potential, though not necessarily immediate, suicide risk. On the other hand, suicide crisis syndrome (SCS) represents a brief, usually rapidly developing, period of high immediate risk for suicide. Impulsivity could be a key factor in SCS translating into actual suicide attempts [5]. The clinical and research challenges in assessing suicidality (suicidal thoughts/behaviors and actions) involve: (i) recognizing the latent high-risk state in SBD and (ii) understanding and preventing the emergence of SCS by assessing impulsivity as a key factor.

Several models to predict the ideations and intentions of suicide have emerged in the past two decades. The interpersonal theory of suicide posits that suicidal desire emerges when individuals experience intractable feelings of perceived burdensomeness and thwarted belongingness. However, ideations do not translate to suicide attempts until an individual has both suicidal desire and capability for suicide, which should also be assessed to model suicidality [6]. Other models of suicide (integrated motivational-volitional model [7], three-step theory [8], and fluid vulnerability theory [9]) face the same conceptual issue in modeling suicidality. Additionally, most of these models are based on self-reported assessments by the individuals, which could be subjective. Clinical characterization for suicidality assessment is also incomplete as it relies on self-reports of dimensions, such as hopelessness [10], when some suicide attempts are rather related to impulsivity (in manic states) or psychosis (e.g., if one has command hallucinations ordering them to end their life). Thus, the current state of knowledge for suicidality prediction is stalled at the following: the prediction of suicide action based on various psychological parameters is promising but not scalable, not generalizable to all populations, and not objective. This is where we introduce impulsivity as a key transdiagnostic measure for suicidality.

Impulsivity is the tendency to act without making a thorough assessment of the situation, lacking pre-action forethought and acting *in the moment*. Impulsivity is a composite, transdiagnostic dimension [11] that combines various behavioral pathways involving emotions aroused by a situation, identifying different possible actions, playing out the consequences of the action in one's brain, and choosing an action (which could include inhibiting a response/delaying action). Thus, impulsivity measurements need to incorporate multiple aspects involved in a behavioral pathway. Currently, the predominant approach to measure impulsivity is using clinical questionnaires, such as Barratt's Impulsiveness Scale (BIS-11) [12], UPPS-P [13], Abbreviated Impulsiveness Scale [14], and Eysenck impulsivity score [15]. These questionnaires are subjective and self-reported by the individuals, so are subject to bias/subjectivity. Towards the objective assessment of impulsivity, behavioral tests and neuro-physiological markers are being investigated. Behavioral tests, such as immediate memory test/delayed memory test (IMT/DMT) [16] and the Flanker test [17] have been developed to assess impulsivity. Heart rate variability (HRV) could also provide a marker of impulsivity [18], specifically during stress since impulsivity mediates an individual's behavior during stress [19]. Similarly, functional brain connectivities, which are associated with a behavioral response, for example, in emotional arousal by a situation and response inhibition, could also serve as impulsivity markers. The insula and prefrontal cortex regions in the brain have been implicated in impulse and emotion processing in suicidality [20]. Brain activation in the limbic system and pre-frontal cortex, either in the task processing stage (when the individual is involved in response inhibition task) or in the resting state, has also been proposed as possible marker of impulsivity/impulsive behaviors [21–24]. Though several studies are considering impulsivity models using objective measurements, analyzing the relation of impulsivity modeling for the specific behavioral correlate with suicidal self-harm has been limited.

Higher impulsivity could be a factor for detrimental behaviors, such as suicidality [25]. Suicidality assesses the risk of suicide in an individual indicated by suicidal behavior, intent, and/or extensive planning of suicide. Some previous studies have investigated impulsivity about suicidality [26–28]. For example, BIS-11 questionnaires were used in a study [28] to assess impulsivity in a bipolar group with suicidality compared with a group without suicidality. The group with suicidality had elevated impulsivity. Similarly, higher impulsivity measures derived from behavioral tests have also been reported in adolescents with suicidality [26]. In one of the studies, functional magnetic resonance imaging (fMRI) activation was employed to obtain *>*90% accuracy in suicidality/non-suicidality classification among 34 participants [29]. However, a follow-up analysis of the same data [30] showed that the unbiased classification accuracy in the study was about 41% only, even below a chance accuracy. Neuroimaging data-based classification showed up to 75% accuracy in another study for suicidality classification [31].

Though impulsivity measures in suicidality have been investigated in previous studies, a multimodal assessment of impulsivity considering the self-report, behavioral, physiological, and neurological basis of impulsivity measures has not been investigated. In this study, we evaluate the impulsivity markers obtained from questionnaires, behavioral tests (IMT/DMT and Flanker tests), HRV features, and resting state (rsfMRI) connectivity in 34 participants with mood disorders [1]. Questionnaire-based impulsivity markers, particularly the BIS were found to assign significantly higher impulsivity scores to the group with higher suicidality. Though the IMT behavioral test too consistently assigned higher impulsivity markers to the suicidality group, there were no significant differences between groups. Similarly, the suicidality group had significantly higher HRV features. Overall, when objective impulsivity markers from multiple modalities were used together in a classifier evaluated in a leave-one-participant-out cross-validation setting, we obtained a suicidality classification accuracy of 96.77% for a three-class suicidality classification. The accuracy obtained was higher than those obtained with a single modality and much better than a baseline classification model, which resulted in 41.93% accuracy. Our study demonstrates the link between impulsivity and suicidality across multiple modalities and is the first reported study on suicidality classification using the multimodal objective impulsivity markers.

## 2. Materials and Methods

### 2.1. Study Group

We enrolled 34 participants with mood disorders, primarily subjects with major depressive disorder (MDD) or bipolar disorder, in our study. The study was approved by the Institutional Review Board (IRB) at Baylor College of Medicine and Harris Health Systems. Informed consent was obtained from the participant, and the study was conducted following ethical guidelines on research with human subjects. The number of male/female participants was 15/19. The average age of the participants was 27.3 ± 5.2 (minimum age: 18 years and maximum age: 35 years). We used the Mini-International Neuropsychiatric Interview (MINI) questionnaire [32] to assess participants' suicidality. The suicidality module of MINI includes 19 questions to assess suicidality (current or lifetime) and criteria relevant to suicide behavior disorder. The suicidality score, indicating the intensity, is categorized as low (1–8 points), moderate (9–16 points), and high/severe (>17 points). Among the participants with a MINI-based suicidality assessment, seven had no suicidality, eleven had low suicidality, four had moderate suicidality, and nine had severe suicidality. Since the number of participants with moderate suicidality was low, we combined the moderate and severe groups to obtain three suicidality groupings of the participants: none (7 participants), low (11 participants), and moderate to severe (13 participants). When suicidality is present, the MINI questionnaire also identifies if the suicidality is current, a lifetime attempt, or likely in the near future. The demographics and groupings on suicidality are summarized in Table 1.

### 2.2. Impulsivity Questionnaires

In this study, we used the 30-item BIS-11 [12] and 59-item UPPS-P [13] questionnaire-based impulsivity assessment. The BIS-11 gives a single impulsivity score characterizing an individual's overall impulsivity, the BIS-total (score range: 30–120). It also provides three subscales representing second-order factors in BIS: *attentional* (8 items), *motor* (11 items), and *nonplanning* (11 items) impulsivity dimensions. UPPS-P, on the other hand, provides five subscales (score range: 1–4, averaged score from constituent questions) corresponding to the following dimensions: *negative urgency*, *premeditation*, *perseverance*, *sensation seeking*, and *positive urgency*. The questionnaire data were available for 33 of the 34 participants; the impulsivity questionnaire was not completed by one participant.

### 2.3. Behavioral Tests

We employed the IMT/DMT [16] and Flanker tests [17] in our study for impulsivity assessment. In the IMT/DMT test, the participants have to respond when the same 5-digit numbers are shown (consecutively in the case of IMT) on the screen and inhibit responses where numbers are different (the most confusing case has numbers differing in a single place). Similarly, in the Flanker test, the participants have to identify the direction of a central left/right arrow, that is flanked by surrounding arrows in a row. We used a test with five arrows. We computed the ratio of the commission error rate to correct detection from the IMT/DMT test as an impulsivity marker [16]. Similarly, we computed the error rate from the Flanker test.

### 2.4. HRV Measurements

We measured the cardiovascular response of the participants under stress using the PulseCam system [33]. The PulseCam system uses a camera and a pulse oximeter to obtain different cardiovascular parameters, such as HRV and perfusion.

We measured the patient's cardiovascular physiology during their stressed state as stress reportedly reveals individual differences in impulsive behaviors, which might not be readily observed in a non-stressed state [19]. The PulseCam measurements were completed for 29 participants. For the remaining five: three participants did not enroll for the PulseCam study component, one participant stopped midway and preferred to discontinue, and there were technical issues during measurements for one participant rendering the data unavailable for analysis. Though both facial video recording and pulse-oximeter measurements are available, in this study, we analyzed the pulse-oximeter measurements only to derive HRV measures. The camera data would be relevant in future analysis where the relation between blood perfusion and impulsivity/stress will be studied. We used the standard Math task and the speech task, part of the Trier social stress test (TSST) stress framework [34] to induce stress in participants. Each stress task was about 5 minutes long.

We used the *biopeaks* toolbox implemented in Python [35] to process the photoplethysmography signal from the pulse oximeter and calculate the following HRV features.

1. Root mean square of the sum of successive differences (RMSSD).
2. Standard deviation of the peak-to-peak intervals (SDRR).
3. Percentage of peak-to-peak intervals lower than 50 ms.
4. Percentage of peak-to-peak intervals lower than 20 ms.

We computed the average HRV features from the stress task phases for participants. HRV differences in groups with different impulsivity have been reported in earlier studies [18, 36]. Suicidal ideation has also been found to be linked with cardiac autonomic dysregulation, which impacts HRV, in a previous study [37].

### 2.5. Functional Magnetic Resonance Imaging

We obtained the fMRI-based functional connectivity of the participant's brain during the resting state and the task phase (while completing the behavioral tests). We used a 3T Siemens Prisma Fit machine for the fMRI scans. In the scope of this study, we analyzed resting state connectivity. We obtained the structural scan with a 1 mm isotropic voxel, echo time of 0.00298 seconds, repetition time of 2.3 seconds, and voxel size of 1 × 1 × 1 mm^3^. Similarly, we obtained the resting state fMRI scans at a resolution of 91 × 109 × 91 with a slice thickness of 2.2 mm, echo time of 0.037 seconds, repetition time of 1 second, and voxel size of 2.2 × 2.2 × 2.2 mm^3^. The fMRI data were processed using the CONN software [38], using the default pre-processing pipeline (97th percentile in normative sample) of CONN to process the functional and structural MRI data.

We used an ROI–ROI analysis in CONN, using the standard Harvard–Oxford and AAL atlas available in CONN defining the brain region parcellations. Emotion and impulse processing have implicated the insula [39–43], particularly its connectivity to the prefrontal cortex (PFC) regions in the context of suicidality [20]. Accordingly, we obtained the connectivity (defined with Fisher transformed correlation of BOLD signal in the atlas regions) between the left and right insula with medial, rostral, and lateral prefrontal cortex regions (MPFC, RPFC, and LPFC, respectively) of the brain atlas.

### 2.6. Multimodal Objective Impulsivity Assessment for Suicidality Classification

We evaluated a multi-class suicidality classifier (none, low, and moderate to severe suicidality classification) using impulsivity measures obtained from different objective modalities (behavioral tests, HRV, and rsfMRI). The classifier was evaluated in a leave-one-participant-out (LOPO) cross-validation setting. We chose LOPO for cross-validation because LOPO approximates clinical deployment [44, 45] and is commonly used for evaluations with a limited number of participants [46–50], as is the case for our study. We used the support vector machine classifier with the radial basis function (RBF) kernel for classification. Support vector machine (SVM) was found to outperform random forest and logistic regression-based classifiers for suicidality prediction. We used the SVM implementation in the *scikit-learn* library of Python with the *gamma* value set to ‘scale' and *C* obtained with cross-validation, using the value of *C* that resulted in the best LOPO classification accuracy when using questionnaire-based features only for prediction. We used mean imputation from the training set within the cross-validation loop to fill in missing features, if any. A multimodal classifier (fusion model) was trained that fused the prediction from three classifiers trained on the objectively measured HRV, behavioral tests, and rsfMRI features. Compared with the early fusion of features, a late fusion of decisions does not encounter increasing dimensionality in a low data setting when different modalities are employed together. We employed a *max-fusion* scheme, that is, prediction_fusionmodel_ = max(prediction_hrv_, prediction_behavioraltest_, prediction_rsfMRI_), which is valid when the target classes are ordinal as is the case in our suicidality prediction. The *max-fusion* scheme was found to outperform early fusion on the feature level and other fusion schemes, such as *min-fusion* where the minimum of all predictions is taken as the final prediction. To evaluate the robustness of the employed classification pipeline, we evaluated the pipeline with (i) label permutation and (ii) random Gaussian signal as input features. These two evaluations are expected to provide near-random classification accuracy and highlight overfitting in the pipeline if any [51]. Additionally, to further assess the robustness of the association between impulsivity features and suicidality, we also evaluated a two-stage classification setup to compare with the proposed direct three-class classification. The two-stage classification consisted of a suicidality/no-suicidality classifier in the first stage and a low/moderate to severe suicidality classifier in the second stage (only invoked if suicidality is predicted in the first stage for a test participant). A high classification would be expected irrespective of the classification pipeline (direct classification or two-stage) if the impulsivity features are predictive of suicidality.

## 3. Results

### 3.1. Group Difference in Terms of Questionnaire-Based Impulsivity

**Figure 1:**
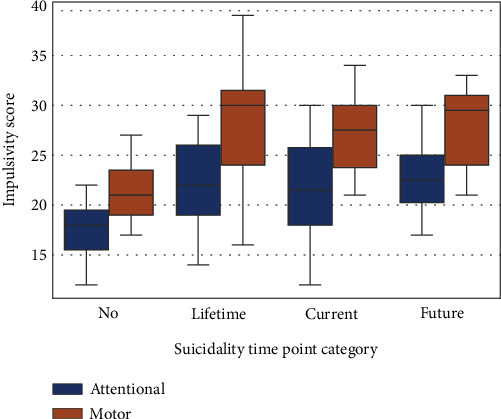
Motor and attentional impulsivity differences based on suicidality timepoint. The motor and attentional impulsivity showed a significant difference between suicidality groups and were thus selected for this visualization to understand if there are impulsivity differences based on suicidality time point categorization.

We computed the impulsivity scores from BIS-11 and UPPS-P, along with the subscales, in the three suicidality groups. The results obtained are shown in Table 2. The groups with higher suicidality had higher impulsivity scores compared with the non-suicidality group for both BIS and UPPS-P questionnaires across subscales. The motor and attentional impulsivity subscale from BIS was significantly different across the suicidality groups (we assessed all group differences using the non-parametric Kruskal–Wallis test). The MINI assessment also produced a timepoint of suicidality, lifetime (*N* = 11), current (*N* = 8), or future (*N* = 8)—a participant can be assigned to multiple time points of suicidality. We assessed the difference between motor and attentional impulsivity based on the suicidality timepoint, as shown in Figure 1. There was no significant difference within the suicidality group (low or moderate to severe) based on the suicidality timepoint.

### 3.2. Behavioral Tests and Impulsivity Questionnaires

We used the Flanker test and IMT/DMT tests to get an objective assessment of impulsive behaviors. Flanker test results from each participant were processed to obtain an error rate. Similarly, commission errors to correct the detection ratio were extracted from the IMT/DMT tests. The average error rate for different suicidality groups is shown in Table 3. The error rates for the suicidality group were higher in the IMT/DMT test and the Flanker test. However, none of the differences were significant.

### 3.3. HRV and Impulsivity

We computed average HRV during the stressed state and assessed the difference between the groups with different suicidality. The results obtained are presented in Table 4. The groups with suicidality had higher HRV compared with the non-suicidality group, and the difference between the groups was significant for the RMSSD, pNN50, and pNN20 features.

### 3.4. Resting State fMRI Connectivity and Impulsivity

We assessed the connectivity between the insula and prefrontal cortex regions using the ROI-to-ROI analysis obtained with CONN toolbox for analyzing fMRI-based brain connectivity. These brain regions are involved in impulse processing about suicidality [20, 52]. The difference between groups in terms of their rsfMRI connectivity is summarized in Table 5. Though there was a difference between groups in terms of the connectivity from the left and the right insula regions, for example, the insula (left) and the left lateral prefrontal cortex connectivity consistently increased with increasing suicidality, the differences were not significant.

### 3.5. Multimodal Impulsivity Assessment for Suicidality Classification

**Figure 2:**
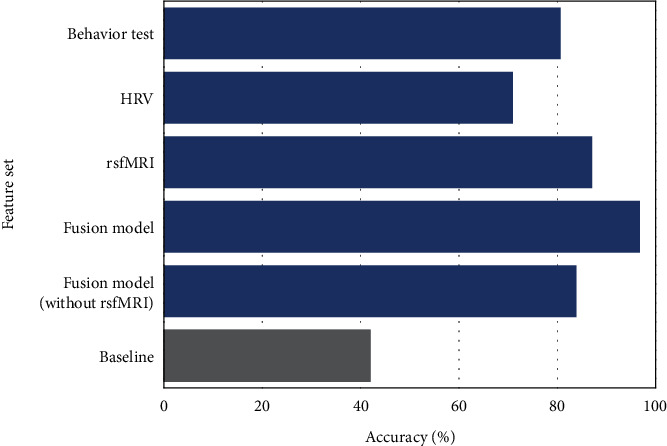
Multiclass classification of suicidality group (none, low, or moderate to severe) using different impulsivity-related markers from objective modalities (behavioral tests, HRV, and rsfMRI). A SVM-based classifier was trained for the classification model. The baseline accuracy of 41.93% is obtained with a model that provides the most common class of the training set as its prediction.

Given the group difference in terms of different impulsivity markers, we evaluated unimodal and multimodal suicidality classifications. The results obtained from an SVM classifier with impulsivity measures from different modalities, and the fusion of objective measures, are shown in Figure 2. A classification accuracy of 96.77% was obtained with the fusion of the multiple objective modalities, which is higher than the classification accuracy obtained with any other single modality and much higher than a baseline accuracy of 41.93% obtained with a model that provides the most common class of the training set as its prediction. The classification accuracy of 96.77% is also higher than the average accuracy of 32.25% obtained under random label permutation and the accuracy of 41.29% obtained when training the model with random Gaussian data as features [51]. The fusion model employed the objectively obtained impulsivity markers from the behavioral tests, HRV, and rsfMRI connectivity. In a fusion model without the rsFMRI-based features, the suicidality classification accuracy obtained was 83.87%. Compared with the direct three-class classifier, the two-stage classifier (first classify if a participant has suicidality, then classify the level of suicidality if the participant was predicted to have suicidality) also resulted in a high classification accuracy of 90.32%.

## 4. Discussion

Suicidality spans the gamut from past suicide attempts to elaborate suicidal planning. Despite the relevance of impulsivity to mental health and specifically self-harm and suicidality, its quantification to characterize suicidality is still elusive. Although questionnaires are most often used for measuring impulsivity, several other objective methods for impulsivity assessment are also being explored to bypass questionnaires' subjective nature. In this study, we pursued a multimodal impulsivity assessment to characterize a clinical population with varying levels of suicidality. Impulsivity markers from questionnaires, behavioral tests, HRV, and rsfMRI were obtained from 34 participants with mental health disorders. Suicidality was assessed using widely used questionnaires (i.e., the MINI as a widely used structured diagnostic instrument) rather than specialized instruments, keeping potential future generalizability and scalability in mind. Our study shows the advantage of multimodal assessments and the inclusion of objective physiological measures and structured interviews in assessments of suicidal propensity. Interestingly, although none of the included subjects were endorsing active suicidal intent, thoughts, or plans at the time of study procedures and follow-up (4 weeks), differences in suicidality and impulsivity classifications persisted, highlighting the possibility of using multimodal impulsivity measures in suicidality assessments in a longitudinal fashion.

BIS and UPPS-P are commonly used questionnaires to assess an individual's impulsivity. In both questionnaires, the groups with suicidality had a higher impulsivity score across all subscales (Table 2), with attentional and motor impulsivity from BIS significantly different across the groups. Higher motor and attentional impulsivity have been associated with higher suicidal risk in previous studies also [53, 54]. The suicidality group had the highest UPPS-P subscale of negative urgency, possibly capturing the group's characteristic to act harshly in the presence of negative stress. From behavioral tests, only the error rates from the IMT test were higher for the suicidality groups, though not significantly so, compared with the non-suicidality group (Table 3). Future studies should investigate other markers derived from behavioral tests that could link to impulsivity differences between groups about suicidality. HRV features have also been linked to impulsivity in previous studies [18, 55]. In our study, the differences in RMSSD, pNN50, and pNN20 features were significant across the suicidality groups. Since HRV could be affected by confounding factors, such as participants' sensitivity to the given stressor, baseline HRV, and demographics, future studies with a larger number of participants should assess approaches to understand suicidality–HRV relations accounting for possible confounders in this relation. Brain connectivity analysis for impulsivity markers has been pursued in previous studies [21–23], implicating insula regions and the pre-frontal cortex regions for impulse control about suicidality [20, 52]. However, the connectivity between the insula and pre-frontal cortex regions did not differ significantly between the groups in our analysis (Table 5). Future studies with a larger participant group could help identify brain regions linked with impulsivity in relation to suicidality. In the current study with a small number of participants, exploring more seed regions in the brain could have elevated the chances of overfitting. Therefore, different seed regions in the pre-frontal cortex and limbic systems implicated in psychopathology should be explored in future studies with a larger number of participants.

We analyzed multiple markers in assessing group differences across suicidality groups. Corrections for multiple comparisons would lead to the observed significant differences falling below the significance threshold. Thus, the markers considered in our analysis could be considered only as weakly associated with suicidality. These weak markers could still be combined to obtain a better predictor of suicidality. To test this possibility, various modalities for impulsivity markers were used in a three-class classification pipeline to predict a given participant's suicidality group (none, low, or moderate to severe). Individually, each modality gave good classification accuracy (>67% for each of the modalities considered). Multiple markers were obtained from each modality. Thus, the combination of markers probably led to a better representation of suicidality group differentiation. This was further highlighted with the fusion of the impulsivity markers from HRV, rsfMRI, and behavioral tests. We obtained a high classification accuracy of 96.77% (Figure 2). The classification accuracy is much higher than the baseline classification accuracy of 41.93% (obtained by always predicting the dominant class in the training set within the LOPO cross-validation) and accuracy obtained under label permutation (32.25% accuracy) or training with Gaussian random data (41.29%). The latter has been proposed as a method to assess classifier overfitting under small dataset scenarios [51]. In a fusion model where the rsfMRI is not included, suicidality prediction accuracy is 83.8%. This indicates that easily deployable measurements, such as behavioral tests and HRV, could together still provide a good suicidality classifier for scalable and more continuous assessments.

A high accuracy (*>*90%) in suicidality classification was reported in an earlier study [29] using fMRI activations alone. However, the evaluation was reported to be biased, and unbiased classification accuracy was reported to be no better than a chance classification [30]. Similarly, another study [31] reported ∼78% accuracy in classifying suicidal/non-suicidal groups using brain imaging modalities. The higher accuracy obtained in our study could be due to the nature of the participant group (participants with mood disorders), indicating the stronger association between suicidality and impulsivity in this particular group, and the diversity of the modality applied for the classification system (rsfMRI modality alone had only 87.09% classification accuracy in our analysis). In future studies with larger and more diverse participants, a suicidality classification could additionally benefit from the availability of other data sources like daily behavioral features and genetic features [3] and needs to be further investigated.

In our analysis, we observed large classification accuracy variations across different classifiers and sensitivity of classification accuracy to the hyperparameters in the chosen SVM model. To get further confidence about the predictive power of the impulsivity features for suicidality prediction, we also evaluated a two-stage classifier where the first classifier assessed if a participant has suicidality (a binary classifier of yes or no), and the second classifier assessed the suicidality level (low or moderate to severe) for participants predicted to have suicidality by the first classifier. We obtained high classification accuracy with the two-stage classifier resulting in a classification accuracy of 90.32% with a fusion of the modalities, hinting at the robustness of the learned classification boundaries that appears across different classification strategies. Previous studies on suicidality prediction using genetic features have also shown remarkable accuracy in suicidality prediction, for example, predicting suicidal ideation within a bipolar disorder group with an area under the curve of 0.98 [56]. Despite the high classification accuracy obtained in our study and previous studies, the question of the generalizability of suicidality prediction on a larger population group remains an open question. A classification pipeline evaluated on a small dataset has risks of overestimation due to overfitting [51]. For our study, a separate validation set from a new study would be helpful to further assess the robustness of the classifier. This will be pursued in future studies. A study with larger and more diverse participants would be further helpful to understand, which classifiers and feature sets could generalize for suicidality prediction in a bigger sample.

Overall, the high suicidality classification accuracy obtained from the impulsivity markers in our study indicates that impulsivity could be a crucial factor in suicidality. In the high suicidality group, higher impulsivity might be a factor in a higher probability of suicidal thoughts and intents translating into action. Studies should be designed in the future to better understand this aspect.

## 5. Conclusions

Although questionnaires are commonly used to assess impulsivity, various objective impulsivity makers based on behavioral tests, HRV, and brain connectivity appear to be useful in the quantification and classification of impulsivity. In this study, we pursued a multimodal assessment of impulsivity to characterize groups with varying levels of suicidality. Questionnaires-based impulsivity subscales and HRV features were significantly different across the suicidality groups. A multimodal objective impulsivity marker-based classifier outperformed the unimodal system in the suicidality/non-suicidality classification of the participants. Impulsivity being a multi-dimensional construct likely benefits from the multimodal representation, which captures various aspects of (neuro-) biological and behavioral pathways involved in impulsive behaviors when it comes to predicting suicidality. Furthermore, analysis of impulsivity's relation to suicidality under different clinical scenarios is needed in future studies, especially analyzing the relation in the presence of strong/periodic negative emotions, substance use, and lethal method availability, to design just-in-time intervention strategies.

## Figures and Tables

**Table 1:** Demographics of participants in our study.

Characteristics	Participants
Number of participants	34
Gender	15 males and 19 females
Age	27.3 ± 5.2
Primary diagnosis	21 MDD
	11 bipolar disorder
	1 panic disorder
	1 post-traumatic stress disorder
Suicidality	7 no suicidality
	11 low suicidality
	13 moderate to severe suicidality
	3 information not provided

*Note*. A total of 34 young participants with mental health disorders were enrolled in the study.

**Table 2:** Group difference in groups with different suicidality in terms of questionnaire-based impulsivity scores.

Impulsivity	Suicidality			
	None	Low	Moderate to severe	Group difference *P*-value
*BIS*				
Total	63.00 ± 12.57	71.91 ± 17.43	79.38 ± 15.32	0.119
Attentional	17.43 ± 3.36	20.55 ± 6.36	23.00 ± 4.12	0.046
Motor	21.43 ± 3.60	26.82 ± 6.97	27.54 ± 4.33	0.044
Nonplanning	24.14 ± 7.31	24.55 ± 5.65	28.85 ± 8.07	0.329
*UPPS-P*				
Negative urgency	2.60 ± 0.78	2.98 ± 0.60	3.21 ± 0.69	0.210
Premeditation	1.84 ± 0.57	2.11 ± 0.59	2.34 ± 0.76	0.295
Perseverance	2.01 ± 0.62	2.34 ± 0.43	2.58 ± 0.50	0.139
Sensation seeking	2.68 ± 0.19	2.82 ± 0.58	2.75 ± 0.75	0.764
Positive urgency	2.03 ± 0.60	2.39 ± 1.01	2.57 ± 1.16	0.618

*Note*. Higher attentional and motor impulsivity were present in the groups with suicidality.

**Table 3:** Group difference between suicidality groups in terms of behavioral test scores obtained from the Flanker and IMT/DMT test.

	Suicidality			
Impulsivity	None	Low	Moderate to severe	Group difference *P*-value
Error rate—Flanker	0.29 ± 0.22	0.19 ± 0.09	0.25 ± 0.16	0.664
Commission error—IMT	0.26 ± 0.04	0.30 ± 0.13	0.41 ± 0.17	0.208
Commission error—DMT	0.60 ± 0.22	0.90 ± 0.97	0.66 ± 0.38	0.934

**Table 4:** Group difference between groups with different suicidality in terms of HRV features obtained with the PulseCam system.

	Suicidality			
Impulsivity	None	Low	Moderate to severe	Group difference *P-*value
SDRR	45.78 ± 22.15	61.08 ± 35.04	82.53 ± 36.97	0.069
RMSSD	40.46 ± 15.87	48.13 ± 31.35	76.43 ± 32.72	0.017
pNN50	16.27 ± 13.25	21.08 ± 17.54	39.95 ± 15.62	0.012
pNN20	37.97 ± 18.17	43.15 ± 23.32	63.34 ± 11.95	0.017

**Table 5:** Group difference of connectivity between the insula and prefrontal cortex regions between the suicidality groups.

	Suicidality			
Impulsivity	None	Low	Moderate to severe	Group difference *P-*value
Insula (L)_MPFC	−0.21 ± 0.33	−0.18 ± 0.25	−0.17 ± 0.44	0.905
Insula (L)_RPFC (L)	0.59 ± 0.12	0.45 ± 0.28	0.55 ± 0.20	0.282
Insula (L)_RPFC (R)	0.48 ± 0.23	0.25 ± 0.26	0.38 ± 0.29	0.186
Insula (L)_LPFC (L)	−0.08 ± 0.26	0.13 ± 0.20	0.18 ± 0.28	0.144
Insula (L)_LPFC (R)	0.03 ± 0.29	−0.17 ± 0.27	0.09 ± 0.25	0.135
Insula (R)_MPFC	−0.15 ± 0.28	−0.30 ± 0.27	−0.10 ± 0.38	0.286
Insula (R)_RPFC (L)	0.52 ± 0.17	0.36 ± 0.23	0.49 ± 0.23	0.259
Insula (R)_RPFC (R)	0.72 ± 0.40	0.38 ± 0.25	0.53 ± 0.22	0.111
Insula (R)_LPFC (L)	−0.24 ± 0.26	−0.02 ± 0.28	0.10 ± 0.27	0.061
Insula (R)_LPFC (R)	0.09 ± 0.30	0.01 ± 0.29	0.19 ± 0.29	0.376
Insula (L)_MPFC	−0.21 ± 0.33	−0.18 ± 0.25	−0.17 ± 0.44	0.905
Insula (L)_RPFC (L)	0.59 ± 0.12	0.45 ± 0.28	0.55 ± 0.20	0.282
Insula (L)_RPFC (R)	0.48 ± 0.23	0.25 ± 0.26	0.38 ± 0.29	0.186

## Data Availability

The clinical data used to support the findings of this study are restricted due to the Baylor College of Medicine IRB regulations to protect patient privacy. Data are available from the corresponding author, Bishal Lamichhane (bishal.lamichhane@rice.edu) for researchers who meet the criteria for access to confidential data.

## References

[1] Moukaddam N., Lamichhane B., Salas R., Goodman W., Sabharwal A. (2023). *Modeling suicidality with multimodal impulsivity characterization in participants with mental health disorder (preprint)*.

[2] Hedegaard H., Curtin S. C., Warner M. (2021). Suicide mortality in the United States, 1999–2019. *NCHS Data Brief*.

[3] Turecki G., Brent D. A., Gunnell D. (2019). Suicide and suicide risk. *Nature Reviews Disease Primers*.

[4] Jordan J. T., McNiel D. E. (2020). Characteristics of persons who die on their first suicide attempt: results from the National Violent Death Reporting System. *Psychological Medicine*.

[5] Klonsky E. D., May A. M. (2015). Impulsivity and suicide risk: review and clinical implications. *Psychiatric Times*.

[6] Joiner T. E. (2005). *Why People Die by Suicide*.

[7] O’Connor R. C., Kirtley O. J. (2018). The integrated motivational–volitional model of suicidal behaviour. *Philosophical Transactions of the Royal Society B: Biological Sciences*.

[8] Klonsky E. D., May A. M. (2015). The three-step theory (3ST): a new theory of suicide rooted in the “Ideation-to-action” framework. *International Journal of Cognitive Therapy*.

[9] Rudd M. D., Ellis T. E. (2006). Fluid vulnerability theory: a cognitive approach to understanding the process of acute and chronic suicide risk. *Cognition and Suicide: Theory, Research, and Therapy*.

[10] Klonsky E. D., Saffer B. Y., Bryan C. J. (2018). Ideation-to-action theories of suicide: a conceptual and empirical update. *Current Opinion in Psychology*.

[11] Sperry S. H., Lynam D. R., Walsh M. A., Horton L. E., Kwapil T. R. (2016). Examining the multidimensional structure of impulsivity in daily life. *Personality and Individual Differences*.

[12] Patton J. H., Stanford M. S., Barratt E. S. (1995). Factor structure of the Barratt impulsiveness scale. *Journal of Clinical Psychology*.

[13] Lynam D. R., Smith G. T., Whiteside S. P., Cyders M. A. (2006). *The UPPS-P: Assessing Five Personality Pathways to Impulsive Behavior*.

[14] Coutlee C. G., Politzer C. S., Hoyle R. H., Huettel S. A. (2014). An abbreviated impulsiveness scale constructed through confirmatory factor analysis of the Barratt impulsiveness scale version 11. *Archives of Scientific Psychology*.

[15] Eysenck H. J., Eysenck S. B. G. (1975). *Manual of the Eysenck Personality Questionnaire (Junior and Adult)*.

[16] Dougherty D. M., Marsh D. M., Mathias C. W. (2002). Immediate and delayed memory tasks: a computerized behavioral measure of memory, attention, and impulsivity. *Behavior Research Methods, Instruments, and Computers*.

[17] Eriksen B. A., Eriksen C. W. (1974). Effects of noise letters upon the identification of a target letter in a nonsearch task. *Perception and Psychophysics*.

[18] Ottaviani C., Zingaretti P., Petta A. M., Antonucci G., Thayer J. F., Spitoni G. F. (2018). Resting heart rate variability predicts inhibitory control above and beyond impulsivity. *Journal of Psychophysiology*.

[19] Raio C. M., Konova A. B., Otto A. R. (2020). Trait impulsivity and acute stress interact to influence choice and decision speed during multi-stage decision-making. *Scientific Reports*.

[20] Schmaal L., van Harmelen A.-L., Chatzi V. (2020). Imaging suicidal thoughts and behaviors: a comprehensive review of 2 decades of neuroimaging studies. *Molecular Psychiatry*.

[21] Li N., Ma N., Liu Y. (2013). Resting-state functional connectivity predicts impulsivity in economic decision-making. *Journal of Neuroscience*.

[22] Brown M. R. G., Benoit J. R. A., Juhás M. (2015). fMRI investigation of response inhibition, emotion, impulsivity, and clinical high-risk behavior in adolescents. *Frontiers in Systems Neuroscience*.

[23] Soloff P. H., Abraham K., Burgess A., Ramaseshan K., Chowdury A., Diwadkar V. A. (2017). Impulsivity and aggression mediate regional brain responses in borderline personality disorder: an fMRI study. *Psychiatry Research: Neuroimaging*.

[24] Kamarajan C., Ardekani B. A., Pandey A. K. (2020). Random forest classification of alcohol use disorder using fMRI functional connectivity, neuropsychological functioning, and impulsivity measures. *Brain Sciences*.

[25] Oquendo M. A., Mann J. J. (2000). The biology of impulsivity and suicidality. *Psychiatric Clinics of North America*.

[26] Kashden J., Fremouw W. J., Callahan T. S., Franzen M. D. (1993). Impulsivity in suicidal and nonsuicidal adolescents. *Journal of Abnormal Child Psychology*.

[27] Horesh N., Gothelf D., Ofek H., Weizman T., Apter A. (1999). Impulsivity as a correlate of suicidal behavior in adolescent psychiatric inpatients. *Crisis: The Journal of Crisis Intervention and Suicide Prevention*.

[28] Mahon K., Burdick K. E., Wu J., Ardekani B. A., Szeszko P. R. (2012). Relationship between suicidality and impulsivity in bipolar I disorder: a diffusion tensor imaging study. *Bipolar Disorders*.

[29] Just M. A., Pan L., Cherkassky V. L. (2017). Machine learning of neural representations of suicide and emotion concepts identifies suicidal youth. *Nature Human Behaviour*.

[30] Verstynen T., Kording K.

[31] Gosnell S. N., Fowler J. C., Salas R. (2019). Classifying suicidal behavior with resting-state functional connectivity and structural neuroimaging. *Acta Psychiatrica Scandinavica*.

[32] Sheehan D. V., Lecrubier Y., Sheehan K. H. (1998). The MINI-international neuropsychiatric interview (M.I.N.I.): the development and validation of a structured diagnostic psychiatric interview for DSM-IV and ICD-10. *Journal of Clinical Psychiatry*.

[33] Kumar M., Suliburk J. W., Veeraraghavan A., Sabharwal A. (2020). PulseCam: a camera-based, motion-robust and highly sensitive blood perfusion imaging modality. *Scientific Reports*.

[34] Birkett M. A. (2011). The Trier Social Stress Test protocol for inducing psychological stress. *Journal of Visualized Experiments*.

[35] Brammer J. C. (2020). Biopeaks: a graphical user interface for feature extraction from heart- and breathing biosignals. *Journal of Open Source Software*.

[36] Mathias C. W., Stanford M. S. (2003). Impulsiveness and arousal: heart rate under conditions of rest and challenge in healthy males. *Personality and Individual Differences*.

[37] Chang H.-A., Chang C.-C., Chen C.-L., Kuo T. B. J., Lu R.-B., Huang S.-Y. (2013). Heart rate variability in patients with fully remitted major depressive disorder. *Acta Neuropsychiatrica*.

[38] Whitfield-Gabrieli S., Nieto-Castanon A. (2012). Conn: a functional connectivity toolbox for correlated and anticorrelated brain networks. *Brain Connectivity*.

[39] Dambacher F., Sack A. T., Lobbestael J., Arntz A., Brugman S., Schuhmann T. (2015). Out of control: evidence for anterior insula involvement in motor impulsivity and reactive aggression. *Social Cognitive and Affective Neuroscience*.

[40] Mortensen J. A., Evensmoen H. R., Klensmeden G., Håberg A. K. (2016). Outcome uncertainty and brain activity aberrance in the insula and anterior cingulate cortex are associated with dysfunctional impulsivity in borderline personality disorder. *Frontiers in Human Neuroscience*.

[41] Chen C.-Y., Yen J.-Y., Wang P.-W., Liu G.-C., Yen C.-F., Ko C.-H. (2016). Altered functional connectivity of the insula and nucleus accumbens in internet gaming disorder: a resting state fMRI study. *European Addiction Research*.

[42] Belin-Rauscent A., Daniel M. L., Puaud M. (2016). From impulses to maladaptive actions: the insula is a neurobiological gate for the development of compulsive behavior. *Molecular Psychiatry*.

[43] Viering T., Hoekstra P. J., Philipsen A. (2021). Functional network topology of the right insula affects emotion dysregulation in hyperactive-impulsive attention-deficit/hyperactivity disorder. *Scientific Reports*.

[44] Koul A., Becchio C., Cavallo A. (2018). Cross-validation approaches for replicability in psychology. *Frontiers in Psychology*.

[45] Saeb S., Lonini L., Jayaraman A., Mohr D. C., Kording K. P. (2017). The need to approximate the use-case in clinical machine learning. *Gigascience*.

[46] Saeb S., Zhang M., Karr C. J. (2015). Mobile phone sensor correlates of depressive symptom severity in daily-life behavior: an exploratory study. *Journal of Medical Internet Research*.

[47] Chikersal P., Doryab A., Tumminia M. (2021). Detecting depression and predicting its onset using longitudinal symptoms captured by passive sensing: a machine learning approach with robust feature selection. *ACM Transactions on Computer–Human Interaction*.

[48] Mullick T., Radovic A., Shaaban S., Doryab A. (2022). Predicting depression in adolescents using mobile and wearable sensors: multimodal machine learning–based exploratory study. *JMIR Formative Research*.

[49] Scangos K. W., Ahmad H. S., Shafi A. (2020). Pilot study of an intracranial electroencephalography biomarker of depressive symptoms in epilepsy. *The Journal of Neuropsychiatry and Clinical Neurosciences*.

[50] Pestian J. P., Grupp-Phelan J., Bretonnel Cohen K. (2016). A controlled trial using natural language processing to examine the language of suicidal adolescents in the emergency department. *Suicide and Life-Threatening Behavior*.

[51] Etienne C., Karim J. (2015). Exceeding chance level by chance: the caveat of theoretical chance levels in brain signal classification and statistical assessment of decoding accuracy. *Journal of Neuroscience Methods*.

[52] Lengvenyte A., Conejero I., Courtet P., Olié E. (2021). Biological bases of suicidal behaviours: a narrative review. *European Journal of Neuroscience*.

[53] Colborn V. A., LaCroix J. M., Neely L. L. (2017). Motor impulsivity differentiates between psychiatric inpatients with multiple versus single lifetime suicide attempts. *Psychiatry Research*.

[54] Doihara C., Kawanishi C., Ohyama N. (2012). Trait impulsivity in suicide attempters: preliminary study. *Psychiatry and Clinical Neurosciences*.

[55] Laird L. K. (2006). *The Effects of Heart Rate Variability, Measures of Impulsivity, and Activity Level in College Students*.

[56] Niculescu A., Levey D., Phalen P. (2015). Understanding and predicting suicidality using a combined genomic and clinical risk assessment approach. *Molecular Psychiatry*.

